# Phenotypic expansion in Zhu‐Tokita‐Takenouchi‐Kim syndrome caused by de novo variants in the *SON* gene

**DOI:** 10.1002/mgg3.1432

**Published:** 2020-07-24

**Authors:** Ryszard Slezak, Robert Smigiel, Malgorzata Rydzanicz, Agnieszka Pollak, Joanna Kosinska, Piotr Stawinski, Maria Malgorzata Sasiadek, Rafal Ploski

**Affiliations:** ^1^ Department of Genetics Wroclaw Medical University Wroclaw Poland; ^2^ Department of Pediatrics and Rare Disorders Wroclaw Medical University Wroclaw Poland; ^3^ Department of Medical Genetics Medical University of Warsaw Warsaw Poland

**Keywords:** intellectual disability, psychomotor delay, *SON* gene, Zhu‐Tokita‐Takenouchi‐Kim syndrome

## Abstract

**Background:**

The genetic etiology of intellectual and psychomotor disability without a defined spectrum of dysmorphic features is usually monogenic. As no diagnostic criteria for such diseases are established, the clinical diagnosis becomes to be a challenge. The object of our paper is to present two patients with non‐specific clinical symptoms for whom whole‐exome‐sequencing identified the new SON mutations and thus allowed for establishing the diagnosis of Zhu‐Tokita‐Takenouchi‐Kim (ZTTK) syndrome. In both patients, the same symptoms including hypotonia, developmental and speech delay, feeding difficulties as well as frequent infections of the respiratory tract and internal ear were observed. However, both cases presented also with exceptional symptoms such as in case 1 ventriculomegaly and asymmetry of ventricles, hypoplastic left heart syndrome (HLHS), intellectual disability, intestinal malrotation, gastroparesis, and duodenal atresia and in the case 2 febrile seizures and reduced IgA levels. We will be presenting the patients and comparing them to 30 previously described cases.

**Methods:**

Whole‐exome sequencing (WES) was performed on the probands’ DNA and paired‐end sequenced (2x100 bp) on HiSeq 1500. Variants considered as disease‐causing were validated in the proband and studied in all available family members by amplicon deep sequencing performed using Nextera XT Kit and sequenced on HiSeq 1500.

**Results:**

We have identified two new variants in SON gene. In case 1 it has been a heterozygous frameshift variant p.(Ala1340GlnfsTer26), while in case 2 it has been a heterozygous frameshift variant, p.(Asp1640GlyfsTer7). Both variants are described for the first time and up to now, are not mentioned in any database.

**Conclusion:**

As there are no precise criteria established for the clinical diagnosis of ZTTK, an identification of *SON* gene mutation by whole‐exome‐sequencing is the best method that allows for a diagnosis of this syndrome.

## Graphical abstract

In the paper, we present both, clinical symptoms as well as newly identified SON variants in our two patients with Zhu‐Tokita‐Takenouchi‐Kim syndrome in relation to clinical and molecular alterations in 30 cases described up to now.

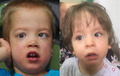

## 1 INTRODUCTION

The etiology of delayed development with intellectual disability in children is heterogeneous. In some cases, the developmental delay is the only clinical handicap, while in others belongs to the spectrum of symptoms of the given syndrome. Thus, in cases presenting with either isolated intellectual disability or with intellectual disability along with only discrete dysmorphic features, the precise diagnosis appears to be a challenge. Introduction of next‐generation sequencing (NGS), allowing for whole‐genome analysis, means a breakthrough in the diagnosis of rare genetic syndrome by opening the possibility of genetic diagnosis, without previous clinical one. Thus, in recent years, many new monogenic diseases/syndromes presenting with either primary or syndromic intellectual disability have been detected, reaching the success rate around 50% (Gilissen et al., 2014; Zhu et al., 2015).

Zhu‐Tokita‐Takenouchi‐Kim syndrome (ZTTK) (MIM #617140) belongs to the group of developmental disorders with heterogeneous clinical presentations, including intellectual disability. ZTTK is inherited as an autosomal dominant trait and results from a mutation in the *SON* gene (Stark et al., 2016).

The *SON* gene (coding for SON DNA binding protein, OMIM 182465) is located on the chromosome 21q22.11. The gene consists of 12 exons. The largest one is the exon 3, constituting 82% of the entire coding region with most of the *SON* variants, which were found in patients with ZTTK, located in this region (Kim et al., 2016; Tokita et al., 2016). All of the described ZTTK cases resulted from de novo *SON* mutations.

The SON plays a key role in the development and is preferentially expressed in undifferentiated stem cells. *SON* haploinsufficiency results in reduced mRNA expression and abnormal RNA splicing of many genes, which are necessary for neural cell migration, metabolic processes, renal and brain development (Ahn et al., 2011; Kim et al., 2019). Thus, it has been proven that SON plays an important role as an RNA splicing regulator with an important role in neurodevelopment.

In patients with ZTTK syndrome, *SON* haploinsufficiency leads to the disturbances of multi‐organ development and thus to multi‐organ defects.

The paper discusses two unrelated Polish patients with ZTTK syndrome in whom de novo heterozygous truncating variants in exon 3 of the *SON* gene were found.

## 2 CLINICAL REPORT

### 2.1 Case 1

**Figure 1:**
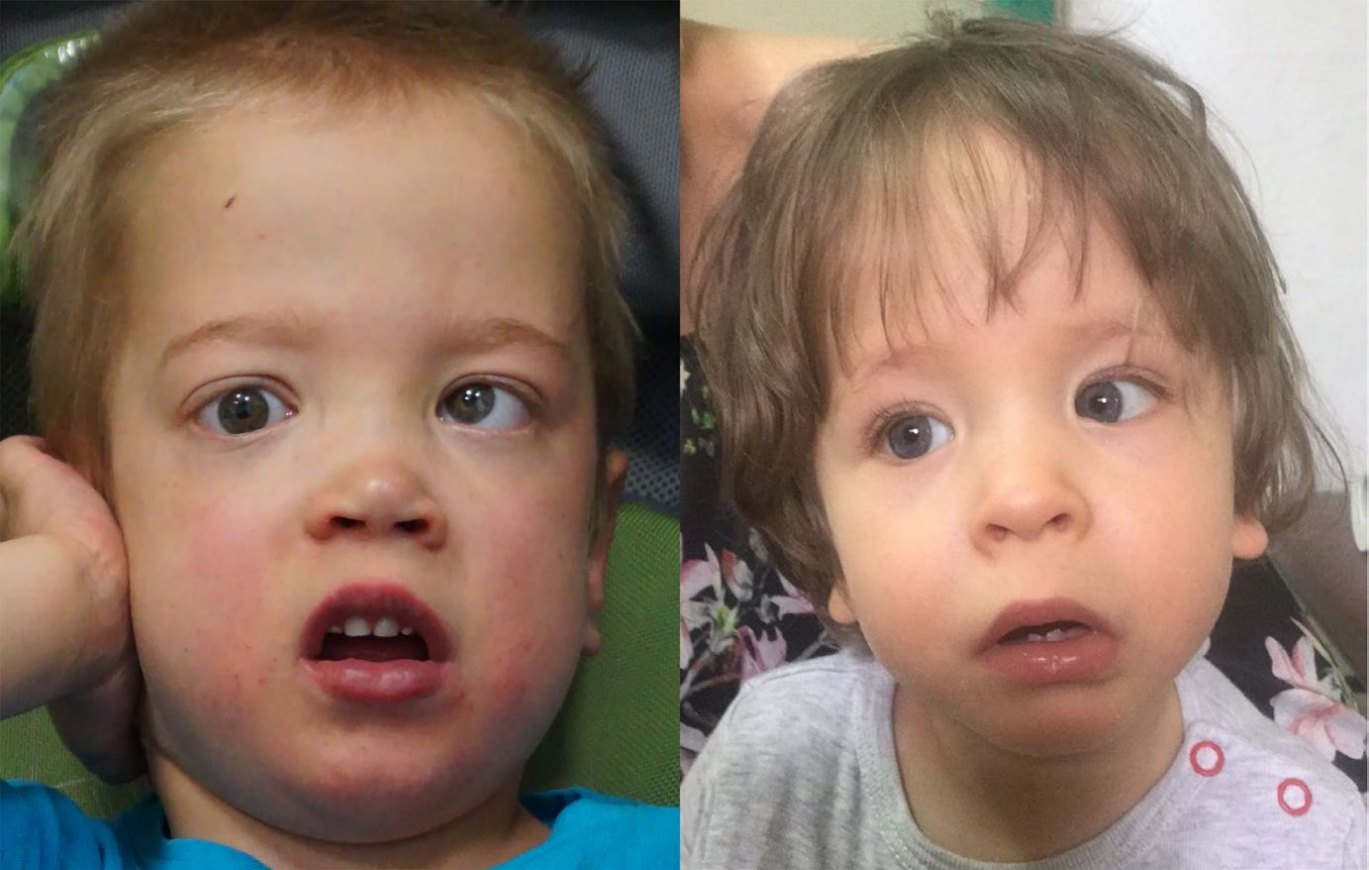
Photos of two new patients with Zhu‐Tokita‐Takenouchi‐Kim syndrome, analyzed in this study

We present a boy, born from the second pregnancy of healthy parents, at 38^th^ week of gestation with birth weight 2540 g, length 53 cm and OFC 32 cm, in good condition (9/9/8/8 Apgar scores). Prenatal ultrasonography at 18 weeks of gestation revealed suspicion of heart defect, and detailed echocardiography of fetal hearft in 21 weeks of gestation confirmed hypoplastic left heart syndrome (HLHS). After birth, HLHS was confirmed in the ultrasonography examination. Moreover, the boy was diagnosed with renal cysts and the horseshoe kidney as well as muscular hypotonia, strabismus, and hyperopia. The hearing screening was normal. In the neonatal period, feeding difficulties, poor suck, duodenal atresia, intestinal malrotation, and gastroparesis were observed. The boy's psychomotor development was significantly delayed: at the age of 2.5, he did not sit alone and said only a single word. His weight was 11 kg (3 percentile), OFC 48.5 cm (10 percentile). Significant hypotonia has been reported. He presented with subtle facial dysmorphia (dolichocephaly, epicanthal folds, thin upper lip vermilion, smooth philtrum, abnormality of dental enamel, wide nasal bridge, wide nasal base, bulbous nose, and abnormality of the pinna. The ultrasonic study of the central nervous system showed ventriculomegaly and asymmetry of ventricles. Thus, at the age of 4.5 years, the body weight was 14 kg (3 percentile) and height was 100 cm (10 percentile). The boy did still not walk alone and his muscle tone was reduced. He did not show distinctive dysmorphic feature but slender fingers, prominent finger pads, joint laxity, hyperconvex nails, pes planus, genu valgus, finger joint hypermobility, sacral dimple, soft, doughy skin, nail dysplasia, and abnormality of the toenails [Figure 1]. Psychologic examination revealed intellectual disability, speech delay, stereotypies (hand flapping), and repetitive compulsive behavior. The patient suffered from frequent upper respiratory tract infections and recurrent otitis media.

### 2.2 Case 2

A 2‐years old male child was the first offspring of healthy, unrelated parents. He was born at 38 weeks of pregnancy with birth weight 2510 g, height 48 cm, and APGAR scores 10. In the neonatal period, generalized hypotonia was observed. The child had no defects in the heart and genitourinary system. He started to sit in his 15^th^ month and walk at 22. In infancy, leading symptoms were feeding difficulties, dysphagia, gastroesophageal reflux, and constipation. At the same time twice, febrile seizures occurred. EEG analysis showed changes in the left mid‐temporal area, activated by sleep. The boy suffered from frequent infections of the upper and lower respiratory tract and ears. Immunological studies showed reduced levels of IgA. Physical examination revealed the following dysmorphic features: short chin, high forehead, fine hair, everted prominent lower lip, epicanthus, downturned mouth, curly hair, broad nasal tip, abnormality of the hairline, long palpebral fissure, shortened nose, frontal bossing, strabismus, thick lower lip, facial asymmetry, hypertelorism, and full cheeks [Figure 1]. At the age of 16 months, his weight was 8 kg (<3 percentile), height 75 cm (5 percentile), OFC 48 cm (77 percentile), and he presented with a delay in speech development; however, intellectual development has been still difficult to evaluate.

## 3 ETHICAL COMPLIANCE

The study was approved by the Institutional Review Board of the Medical University of Wroclaw and written informed consent was obtained from the parents of all pediatric patients included in this study.

## 4 GENETIC TEST—METHODS AND RESULTS

Before whole‐exome sequencing (WES), the following genetic tests were performed yielding normal results: karyotype, as well as array CGH. Array‐CGH assay was performed in both patients and any structural chromosomal aberration was revealed (Agilent SurePrint G3 CGH ISCA v2, 8x60 K).

Whole‐exome sequencing (WES) was performed on the probands’ DNA using SureSelect Human All Exon v5—Case 1 or Human All Exon v7—Case 2 (Agilent Technologies, Palo Alto, CA, USA) and paired‐end sequenced (2x100 bp) on HiSeq 1500 (Illumina, San Diego, CA, USA). Bioinformatics analysis of WES raw data was performed as previously described (ref: PMID: 30254215). Variants considered as disease‐causing were studied in the proband and all available family members by amplicon deep sequencing performed using Nextera XT Kit (Illumina) and HiSeq 1500 (Illumina).

In Case 1, we identified a heterozygous frameshift variant in *SON* gene NC_000021.9(NM_138927.3):c.4018delG, p.(Ala1340GlnfsTer26)) which results in premature termination codon, thus predicted to cause the loss of the full‐length 2426 amino acids protein due to truncation after first 1365 residues. In Case 2, we identified a heterozygous frameshift variant in the *SON* gene (NC_000021.9(NM_138927.3):c.4919_4923delACATG, p.(Asp1640GlyfsTer7)) which results in premature termination codon thus predicted to cause loss of the full‐length 2426 amino acids protein due to truncation after first 1646 residues.

Both identified variants c.4018delG and c.4919_4923delACATG have a null frequency in all tested databases (including gnomAD https://gnomad.broadinstitute.org/ and an in‐house database of >2000 Polish individuals examined by WES).

We did perform a kinship confirmation based on the STR analysis. The family study showed the absence of examined variants in probands’ healthy parents and sibling indicating de novo events. Moreover, both variants are predicted to subject resultant *SON* transcripts to degradation by nonsense‐mediated decay (based by NMDEscPredictor [ref: PMID: 30032986]), thus reduction of *SON* expression level is expected. According to the recommendations of the American College of Medical Genetics and Genomics (ACMG) [PMID:25741868], both detected *SON* variants (c.4018delG and c.4919_4923delACATG) were classified as likely pathogenic.

## 5 DISCUSSION

Thirty children with ZTTK syndrome have been described so far. Twenty of them were presented by Kim et al. (2016), seven by Tokita et al. (2016), and one case was described by Takenouchi, Miura, Uehara, Mizuno, and Kosaki (2016) and Yang, Xu, Yu, Huang, and Yang (2019). Moreover, Zhu et al. (2015) detected a SON mutation in a 10‐month‐old child with psychomotor developmental delay, when testing the group of 113 patients with non‐characteristic developmental disorders and their healthy parents (trios) using next‐generation sequencing. On the basis of their observations, the pattern of most characteristic features for the ZTTK syndrome has been established.

Here, we present two patients with symptoms similar to those described by other authors as shown in Table 1 and in whom the pathogenic variants in the *SON* gene were identified. In both patients we found new, heterozygous frameshift variants in the *SON* gene which results in premature termination codon, thus predicted to cause loss of the full‐length amino acids due to truncation protein.

**Table 1:** Clinical characteristics of patients with *SON* gene mutations

Clinical abnormalities	Patient 1	Patient 2	Zhu et al. (2015)	Tokita et al. (2016)	Kim et al. (2016)	Takenouchi et al. (2016)	Yang et al. (2019)	Summary
Motor delay	1/1	1/1	1/1	ND	ND	1/1	1/1	5/5
Speech delay	1/1	1/1	ND	ND	ND	1/1	ND	3/3
Global development delay	1/1	1/1	1/1	7/7	20/20	1/1	1/1	32/32
Short stature	0/1	0/1	ND	5/7	10/20	1/1	1/1	17/31
Generalized Hypotonia	1/1	1/1	ND	5/6	15/20	1/1	ND	23/29
Intellectual disability	1/1	ND	1/1	ND	20/20	1/1	1/1	24/24
Age at birth >38 Hbd	1/1	1/1	ND	2/7	11/19	1/1	1/1	17/30
OFC <2SD	0/1	0/1	ND	3/7	4/19	0/1	ND	7/27
Low birth weight (<2SD)	1/1	1/1	ND	5/6	8/20	0/1	1/1	16/30
Seizures	0/1	1/1	1/1	2/6	11/20	0/1	0/1	15/31
EEG abnormalities	0/1	1/1	ND	ND	ND	0/1	ND	1/3
Developmental regression	0/1	0/1	ND	3/7	ND	ND	ND	3/9
Brain abnormalities	1/1		1/1	5/6	17/19	0/1	1/1	25/29
Ventriculomegaly	1/1		0	3/6	14/19	0/1	1/1	19/29
Abnormality of corpus callosum	ND		0	2/6	10/19	0/1	1/1	13/27
White‐matter abnormalities	ND		1/1	0/6	4/19	0/1	1/1	6/28
Cerebellar abnormalities	ND	ND	0	0/6	4/19	0/1	0/1	4/27
Behavioral Problems	1/1	1/1	ND	ND	ND	ND	ND	2/2
Autism	0/1	0/1	ND	3/5	3/20	ND	ND	6/27
Short philtrum	0/1	0/1	ND	4/7	0/1	ND	1/1	5/11
Smooth philtrum	1/1	0/1	ND	3/7	0/1		ND	4/10
Thin lips	1/1	0/1	ND	5/7	0/1		1/1	7/11
Epicanthal fold	1/1	1/1	ND	1/7	1/1		ND	4/10
Full cheeks	0/1	1/1	ND	2/7	1/1		ND	4/10
Prominent forehead/frontal bossing	0/1	1/1	ND	0/7	0/1		1/1	2/11
Macrocephaly	0/1	0/1	1/1	ND	1/1		ND	2/4
Depressed/wide nasal bridge	1/1	1/1	ND	3/7	1/1		1/1	7/11
Downslanting palpebral fissures	0/1	1/1	ND	5/7	ND		ND	6/9
Abnormality of heart	1/1	0/1	1/1	2/5	5/20	1/1	0/1	10/30
HLHS	1	0	0	0	0	ND	0	1
PDA/ASD/VSD	0	0	1	2	4	ND	0	7
Other	0	0	0	0	1	1	0	2
Abnormality of Urogenital System	1/1	0/1	ND	5/5	6/20	0/1	ND	12/28
Horseshoe/dysplastic kidney	1	0		1	2	0		4
Unilateral kidney agenesis	0	0		0	0	0		0
Renal cysts	1	0		0	1	0		2
Inguinal hernia	0	0		1	1	0		2
Respiratory problems	0/1	0/1	ND	5/7	1/20	0/1	1/1	7/31
Skeleton abnormality	1/1	0/1	ND	6/7	17/20	0/1	1/1	25/31
Join hypermobility	1	0		3	9	1	0	14
Scoliosis/kyphosis	0	0		1	3	0	1	5
Hemivertebrae	0	0		1	2	0	0	3
Contractures	0	0		0	2	0	0	2
Gastrointestinal abnormalities	1/1	1/1	1/1	7/7	3/20	ND	ND	13/30
Feeding difficulties in infancy	1/1	1/1	ND	7/7	13/19	1/1	ND	23/29
Abnormality of the skin/hair/nails	1/1	0/1	ND	ND	1/20	ND	1/1	3/23
Sacral dimple	1/1	0/1	ND	ND	1/20	ND	1/1	3/23
Abnormalities of immunological system	1/1	1/1	ND	3/7	3/20	ND	ND	8/29
Recurrent otitis media	1/1	1/1	ND	ND	2/20	ND	ND	4/22
Abnormal vision	1/1	0	ND	5/6	15/20	ND	ND	21/28
Strabismus	1/1	1/1	ND	4/6	11/20	ND	ND	17/28
Cortical visual impairment	0/1	0/1	ND	2/6	4/20	ND	ND	6/28
Mild hearing impairment	0/1	0/1	ND	ND	2/20	ND	ND	2/22
Craniosynostosis	0/1	0/1	ND	ND	3/20	ND	ND	3/22

Abbreviation: ND, no data.

Zhu‐Tokita‐Takenouchi‐Kim syndrome caused by *SON* mutation is characterized by congenital hypotonia and developmental delay as well as intellectual disability with behavioral problems. Central nervous system defects were found in 86% of patients. Most often it was the widening of the ventricular system and abnormalities of corpus callosum. Seizures were observed in more than half of the patients; however, EEG changes were confirmed in one‐third of them. A much higher percentage of children presented with behavioral disorders, including autism (observed in about 20% of cases). All examined children had varying degrees of intellectual disability.

ZTTK syndrome should be taken into account in neonates presenting with intrauterine growth retardation (IUGR), congenital brain, heart and genitourinary defects, and feeding difficulties. Most of the children were born before 38 weeks of pregnancy. In childhood, half of them presented with growth deficiency. In infancy, growth and weight disturbances are evident due to feeding difficulties, dysphagia, poor suck, gastroesophageal reflux, either chronic diarrhea or constipation. In this period, an increased frequency of infections, especially of middle ear, urinary, and respiratory tracts (lower and upper respiratory tract) are also observed, probably resulting from a deficiency of IgG and IgA immunoglobulins (Kim et al., 2016; Tokita et al., 2016). One‐third of patients were diagnosed with a variety of heart defects among which patent ductus arteriosus (PDA), atrial septum (ASD) or ventricular septum (VSD) defects were most frequently observed. Those clinical symptoms are often accompanied by skeletal system defects such as flat feet, scoliosis, kyphoscoliosis, bone age delay, hemivertebrae, pectus excavatum, rib fusion, craniosynostosis, a short toe, cervical cord compression, abnormality of limb bone morphology, hip dislocation, genu valgum, lumbar hyperlordosis, disproportionate short‐trunk stature, posterior fusion of lumbosacral vertebrae, thoracic hemivertebrae, short fingers, aplasia/hypoplasia of the thumb, a short distal phalanx of finger, small hand, and abnormal pelvis bone morphology. Concomitant disorders of connective tissue results in joints laxity and hypermobility, contractures, arachnodactyly, syndactyly (2/3 toe) as well as hyperextensibility of the elbow. Abnormalities of the urogenital system were found in 7 of 23 examined patients with horseshoe or hypoplastic kidney most frequently observed. Eye disorders have also been reported in 21 out of 28 patients. Strabismus was the most frequently described change.

When comparing our patients with those presented by other authors, both similarities and dissimilarities in their clinical symptoms were observed. In the two cases presented by us, the birth occurred at the expected date, birth weight was low and growth deficiency has been observed in their childhood. Both of them presented with the unique symptoms such as elevated head circumference in childhood as well as the hypoplastic left heart in one of them.

The facial dysmorphic features of our cases seem to be similar to those described by other authors (see Figure 1). The most common are: facial asymmetry, downslanded palpebral fissures, deeply set eyes, blue sclera's, epicanthus, low set ears, short and smooth philtrum, thin upper lip vermilion, wide and depressed nasal bridge and wide, a bulbous tip of the nose with prominent nares. Less frequently changes such as synophrys, lateral or horizontal sparse eyebrows, unilateral ptosis, narrow mouth, cleft palate, oligodontia, full cheeks, downturned corners of the mouth, and abnormality of the pinna were noted. Moreover, no developmental regression, hearing impairment, and respiratory problems were observed in our patients, as it has been described in cases published by other authors.

Since these symptoms of presented ZTTK patients are not very distinctive, hence diagnostics employing standard clinical methods are in practice useless.

Concluding, ZTTK syndrome is an example of a disease in which diagnosis is possible only by whole‐exome sequencing, because of extensive clinical and genetic heterogeneity. Moreover, as mutations in *SON* may also explain a part of cases of apparently isolated intellectual disability, we suggest to include *SON* into the clinical panel of genes associated with developmental and intellectual disability, testing by the NGS method.

## CONFLICTS OF INTEREST

The authors report no conflicts of interest. The authors alone are responsible for the content and writing of this article. Written informed consent was obtained from the patient for the publication of this case study and any accompanying images.

## AUTHORS’ CONTRIBUTIONS

Conception of the work: SR and SR; Data collection: SR and SR; Data analysis and interpretation: SR, SR, RM, PA, KJ, SP, and PR; Drafting the article: SR, SR, SM; Critical revision of the article SM and PR. All authors reviewed the results and contributed to the final manuscript.
